# Effects of percutaneously-implanted epidural stimulation on cardiovascular autonomic function and spasticity after complete spinal cord injury: A case report

**DOI:** 10.3389/fnins.2023.1112853

**Published:** 2023-02-16

**Authors:** Ashraf S. Gorgey, Jacob Goldsmith, Ahmad Alazzam, Robert Trainer

**Affiliations:** ^1^Spinal Cord Injury and Disorders Center, Hunter Holmes McGuire VA Medical Center, Richmond, VA, United States; ^2^Department of Physical Medicine and Rehabilitation, Virginia Commonwealth University, Richmond, VA, United States; ^3^Physical Medicine and Rehabilitation, Hunter Holmes McGuire VA Medical Center, Richmond, VA, United States

**Keywords:** percutaneous epidural stimulation, autonomic nervous system, spasticity, spinal cord injury, exoskeleton, rehabilitation

## Abstract

**Importance:**

There is a revived interest to explore spinal cord epidural stimulation (SCES) to improve physical function after spinal cord injury (SCI). This case report highlights the potential of eliciting multiple functional improvements with a single SCES configuration, a strategy which could improve clinical translation.

**Objective:**

To determine whether SCES intended to facilitate walking also acutely yields benefits in cardiovascular autonomic regulation and spasticity.

**Design:**

Case report from data collected at two timepoints 15 weeks apart from March to June 2022 as part of a larger clinical trial.

**Setting:**

Research lab at Hunter Holmes McGuire VA Medical Center.

**Participant:**

27-year-old male, 7 years post a C8 motor complete spinal cord injury.

**Intervention:**

A SCES configuration intended to enhance exoskeleton-assisted walking training applied for autonomic and spasticity management.

**Main outcomes and measures:**

The primary outcome was cardiovascular autonomic response to a 45-degree head-up-tilt test. Systolic blood pressure (SBP), heart rate (HR), and absolute power of the low-frequency (LF) and high-frequency (HF) components of a heart-rate variability analysis were collected in supine and tilt with and without the presence of SCES. Right knee flexor and knee extensor spasticity was assessed *via* isokinetic dynamometry with and without SCES.

**Results:**

At both assessments with SCES off, transitioning from supine to tilt decreased SBP (assessment one: 101.8 to 70 mmHg; assessment two: 98.9 to 66.4 mmHg). At assessment one, SCES on in supine (3 mA) increased SBP (average 117 mmHg); in tilt, 5 mA stabilized SBP near baseline values (average 111.5 mmHg). At assessment two, SCES on in supine (3 mA) increased SBP (average 140 mmHg in minute one); decreasing amplitude to 2 mA decreased SBP (average 119 mmHg in minute five). In tilt, 3 mA stabilized SBP near baseline values (average 93.2 mmHg). Torque-time integrals at the right knee were reduced at all angular velocities for knee flexors (range: −1.9 to −7.8%) and knee extensors (range: −1 to −11.4%).

**Conclusions and relevance:**

These results demonstrate that SCES intended to facilitate walking may also enhance cardiovascular autonomic control and attenuate spasticity. Using one configuration to enhance multiple functions after SCI may accelerate clinical translation.

**Clinical trial registration:**

https://clinicaltrials.gov/ct2/show/, identifier NCT04782947.

## Highlights

– Question: Can spinal cord epidural stimulation (SCES) intended to facilitate walking benefit autonomic function and spasticity in a person with spinal cord injury?
– Findings: In this case report, a SCES configuration that yielded enhanced gait in an exoskeleton also abolished a drop in blood pressure during orthostatic challenge and attenuated spasticity in one participant with a motor complete C8 spinal cord injury.
– Meaning: Current research suggests mapping for individual SCES configurations is necessary for specific target functions – however, this approach may be difficult to translate to the clinic. The potential of individual SCES configurations to improve multiple functions warrants further exploration.

## Introduction

Spinal cord injury (SCI) can result in complications such as low blood pressure and muscle spasticity. An emerging strategy to improve function after SCI is spinal cord epidural stimulation (SCES). SCES has emerged as an experimental therapy that can facilitate restoration of motor control in persons with SCI. The potential of SCES to reduce spasticity has been known for over 40 years (Richardson et al., 1979). SCES was previously used to manage spasticity after Multiple Sclerosis, and this eventually resulted in improved motor function (Cook, 1976). SCES was also identified to alleviate spasticity (Barolat et al., 1988) and resulted in improved voluntary mobility across the knee or ankle joints in persons with SCI, further suggesting its utility in enhancing motor outcomes in persons with upper motor neuron injuries (Dimitrijevic et al., 1986).

Recently, SCES has shown potential to improve multiple functions, including overground ambulation (Harkema et al., 2011; Angeli et al., 2014, 2018; Sayenko et al., 2014; Rejc et al., 2015; Gill et al., 2018; Wagner et al., 2018) and regulation of low blood pressure (Aslan et al., 2018; Harkema et al., 2018; West et al., 2018; Darrow et al., 2019). Previous reports indicated SCES effectively regulated the sympathetic activity to attenuate orthostatic intolerance and regulate blood pressure in persons with SCI (Aslan et al., 2018; Harkema et al., 2018; West et al., 2018; Darrow et al., 2019; Ditterline et al., 2020; Squair, 2021). Furthermore, the mechanisms for regulating hemodynamic challenges after SCI has been recently highlighted in rodents, primates and humans with SCI (Squair, 2021). We have recently shown that 14 weeks of body weight supported treadmill training with SCES, resulted in normalized unstable resting seated blood pressure, increased the number of training bouts per session, and decreased the percentage of body weight support to 69% (Gorgey et al., 2022). The aforementioned studies focused primarily on studying paddle implantation with limited evidence about the feasibility of using implanted percutaneous SCES to enhance autonomic functions in persons with SCI. Paddle implantation provides more spatial coverage and stability, but also require a more intensive surgery as spinal laminectomy or laminotomy. On the contrary, percutaneous implantation may be considered a less invasive approach and may involve immediate access to the epidural space, but the leads may potentially migrate (Kim et al., 2011).

**FIGURE 1:**
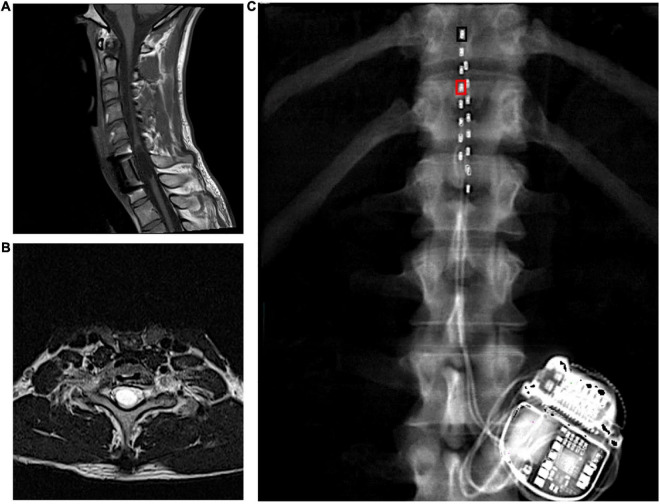
**(A)** Longitudinal and **(B)** axial T2 MRIs highlighting the level of injury in a person with C8 complete motor and sensory SCI that were captured prior to implantation. **(C)** Leads placements with inter-pulse generator as captured by dual energy x-ray absorptiometry of the spine region. The black rectangle denotes the placement of the cathode at 0 and the right rectangle denotes the placement of the anode at 3. These configurations (–0 and +3) yielded rhythmic locomotor-like activity. The leads were staggered where the left lead covered the mid-line of T11 vertebral body to proximal border of L1 and the right lead extended from the T11/T12 inter-vertebral space to the mid-line of L1 vertebral body.

To date, reports show that individual SCES configurations – i.e., electrode arrangement and stimulation parameters – are needed to enable individual functions (Angeli et al., 2014; Sayenko et al., 2014; Rejc et al., 2015; Darrow et al., 2019). In fact, a defining characteristic of SCES configurations to enhance cardiovascular autonomic function is to not induce any lower extremity muscle activity (Aslan et al., 2018; Harkema et al., 2018; West et al., 2018). Identifying optimal person-specific and function-specific SCES configurations is labor-intensive and time-consuming (Angeli et al., 2014; Sayenko et al., 2014; Rejc et al., 2015). Pursuant to this, the increasing occurrence of doctors performing off-label implantations (Gorgey and Gouda, 2022) or persons with SCI electing to self-pay for a SCES system implantation has yielded more variable results than those from research laboratories dedicated to optimizing SCES for persons with SCI (Gorgey et al., 2020). One potential clinically feasible strategy to maximize functional benefits from SCES would be determining if SCES configurations intended for one function yield benefits for other functions or bodily systems. Previous reports by other groups clearly showed that stimulation targeting motor control could have beneficial off target outcomes on blood pressure and body composition (Aslan et al., 2018; Beck et al., 2021). Herein, we report the beneficial, off-target effects of percutaneously implanted SCES configuration intended for overground stepping training on blood pressure and spasticity in 27-year-old male following 7 years post a sensory and motor complete C8 SCI as result of fall (Figures 1A, B).

## Materials and methods

The participant received an implanted a pulse generator with two eight-electrode percutaneous SCES leads (Intellis Epidural Stimulator, Medtronic, Minneapolis, USA) between the T11-L1 vertebrae to cover the lumbosacral enlargement (Figure 1C) as part of an institutional review board (IRB)-approved, registered clinical trial (NCT04782947; IDE# G190003).

### Implantation of percutaneous SCES

This is a 2-step process where temporary implantation precedes permanent implantation. The SCES system (Intellis Epidural Stimulator, Medtronic, Minneapolis, USA) was used to electrically stimulate the lumbosacral enlargement. During temporary implantation, two 8-electrode lead arrays were implanted utilizing fluoroscopic guidance to extend between T11-L1 vertebral bodies. Prior to both temporary and permanent procedures Hibiclens^®^ (chlorhexidine) soap skin cleanser and Bactroban^®^ (mupirocin) 2% ointment were provided for 7 days to reduce bacterial colonization of the participant’s skin. An anesthesia preoperative evaluation was performed, and consent obtained prior to entrance into the operating room. After local anesthetic injection with lidocaine, 14-gauge epidural sterile needles were used using x-ray guidance and loss of resistance technique to access the epidural space at the L2/L3 intervertebral space. The leads were then threaded in the epidural space and the configuration (i.e., stimulation parameters at 2 Hz) were set to evoke motor potentials as indicated by visible motor contractions of the paralyzed muscles. The leads were then taped and glued to the skin after being connected to the external stimulator and remained for 5 days before explantation of the temporary leads.

Four weeks later, the participant underwent permanent implantation with monitored anesthesia care sedation in the operating room. After accessing the epidural space with 2 separate 14-gauge epidural needles, the leads were then navigated in the epidural space to the right and left sided final positions. The pulse generator was then placed after creating a pocket between the muscles and skin in the participant’s lower back between the iliac crest and the 12th rib, ipsilateral to the incision site (Figure 1C). Following hemostasis, the wound was closed in 2–3 layers, dermabond, occlusive dressing and tape were placed over the wound. A belly band was provided for patient comfort.

The process was split into temporary and permanent implantation to ensure appropriate placement of the leads to accurately elicit motor function. Additionally, the temporary implantation may reveal possible unanticipated emerging medical events and may lead to withdrawal from the study. Furthermore, the participant may deny participation because of feeling of discomfort or pain following temporary implantation and may decide to withdraw before undergoing permanent implantation.

### SCES mapping configuration

**FIGURE 2:**
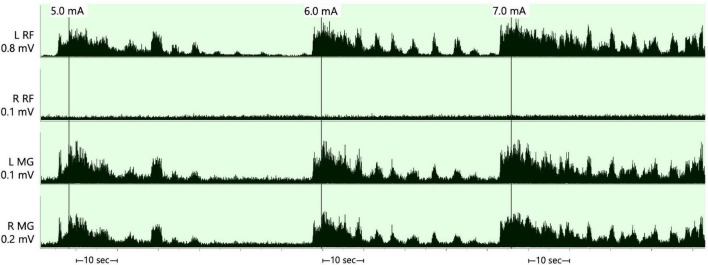
Supine rhythmic electromyography (EMG) activities of the left and right rectus femoris (RF) and medical gastrocnemius muscles initiated when the stimulation parameters were set at a frequency of 25 Hz, pulse duration of 250 μs and amplitude of the current that progressed from 5.0–7.0 mA after configuring the cathode at –0 and the anode at +3. The rhythmicity pattern resulted in periods of EMG bursts that followed by periods of relaxation in three out of the four muscle groups and was less obvious in the right rectus femoris muscle. The EMGs presented are rectified and bandpass filtered at 10–990 Hz. RF, rectus femoris; MG, medial gastrocnemius; mV, millivolts; mA, milliamps; sec, seconds.

The SCES mapping was approximately conducted after 6 months from permanent implantation. The detailed process of establishing rhythmic, locomotor-like activity was previously described by our laboratory (Gorgey and Gouda, 2022). We attempted to identify the cathodal-anodal locations that yield electromyography (EMG) activities of the rectus femoris and then followed by medial gastrocnemius (GM) muscles as previously described (Minassian et al., 2004; Gorgey and Gouda, 2022). These configurations were tested at 2 Hz at three different pulse durations (250, 500, and 1,000 μs) and after ramping the current from 1 to 10 mA. After selection of these configuration, the frequency that yielded rhythmic-locomotor-like activity was then determined (Figure 2).

A SCES configuration that yielded rhythmic, locomotor-like activity in supine was subsequently used to enhance gait during exoskeleton-assisted walking (EAW) training – detailed results are presented elsewhere (Gorgey et al. unpublished work; Figure 2). This configuration was set at −0 (cathode) and +3 (anode) and adjusted at frequency of 25 Hz, pulse duration of 250 μs and amplitude of current at 3 mA (Figure 1C).

### Rehabilitation intervention

**FIGURE 3:**
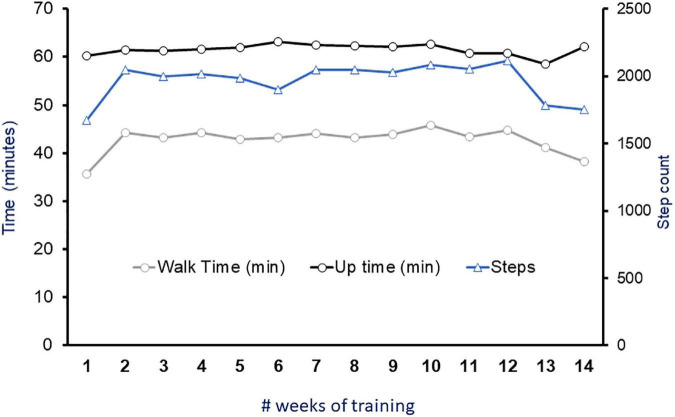
Fourteen weeks of exoskeleton walking training. Each week consisted of an average of three sessions per week. Circles represent time (minutes) for average total walk time and uptime. Triangles represent average total number of steps achieved throughout each week of training. During this period, exoskeleton was set at a variable assist mode to enable stepping with SCES on during 60 min of EAW.

Participant was enrolled in EAW training for 3× per week during the two-assessment periods. The EAW training was performed as part of his weekly training program to facilitate motor recovery for approximately 60 min. To accomplish this goal, an exoskeleton (EksoNR, Ekso Bionics, CA, USA) was set at variable assistance mode that allowed the participant to integrate his volitional effort *via* SCES with EAW (Gorgey et al., 2020). The minimum assistance provided during EAW reflected his volitional attempt enabling stepping during walking. A detailed performance of the participant as indicated by walking time (min), up time (min) and number of steps were highlighted (Figure 3).

During this period, participant was also engaged in 60 min task specific training for 3× per week that focused on sit-to-stand activity, Romanian deadlift for trunk control either through using a standard walker or standing frame. The goal of this training period is to enhance his ability to allow him to enable trunk control and secure independent standing with SCES on. Two or more research assistants were commonly involved during the training to provide necessary physical support when needed.

**FIGURE 4:**
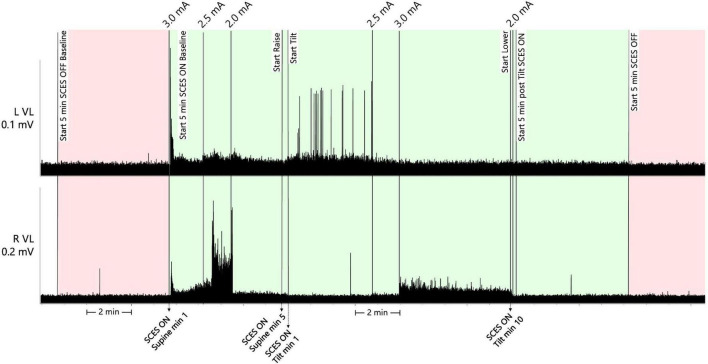
Raw EMG activities of the left and right vastus lateralis muscles for 5 min with SCES off in supine position (pink), followed by 5 min in supine and 10 min tilt with SCES on (both green). The participant was then tilted back to supine position and the SCES remained on for another 5 min (green) followed by another 5 min with the SCES off (pink). The EMG activities were captured only during the second assessment period. min, minutes; mV, millivolts; mA, milliamps; SCES, spinal cord epidural stimulation.

After 4 weeks of EAW training with the rhythmic, locomotor -like activity configuration, the participant self-reported reduced symptoms of low blood pressure when using this configuration outside of training in his daily life. Thus, an interim assessment of the acute effects of this configuration (hereon referred to as “EAW enhancement SCES”) on systolic blood pressure (SBP), heart rate (HR), and low-frequency (LF) and high-frequency (HF) components of a heart rate variability analysis to a 45-degree head-up-tilt test was conducted. Beat-to-beat SBP and HR, and a five-lead echocardiogram (for heart rate variability analysis) were recorded non-invasively (Finapres NOVA, Enschede, the Netherlands). Following this, the participant also self-reported that use of EAW enhancement SCES attenuated his spasticity. 15 weeks after the first interim assessment when regularly scheduled study assessments took place, the acute effects of EAW enhancement SCES on spasticity were tested by measuring passive torques on an isokinetic dynamometer (Gorgey et al., 2011) (Biodex Shirley, NY), and the effects of a head-up-tilt test on SBP, HR, LF, and HF were re-tested and the EMG activities of the right and left vastus lateralis muscles were recorded (Figure 4).

## Results

**FIGURE 5:**
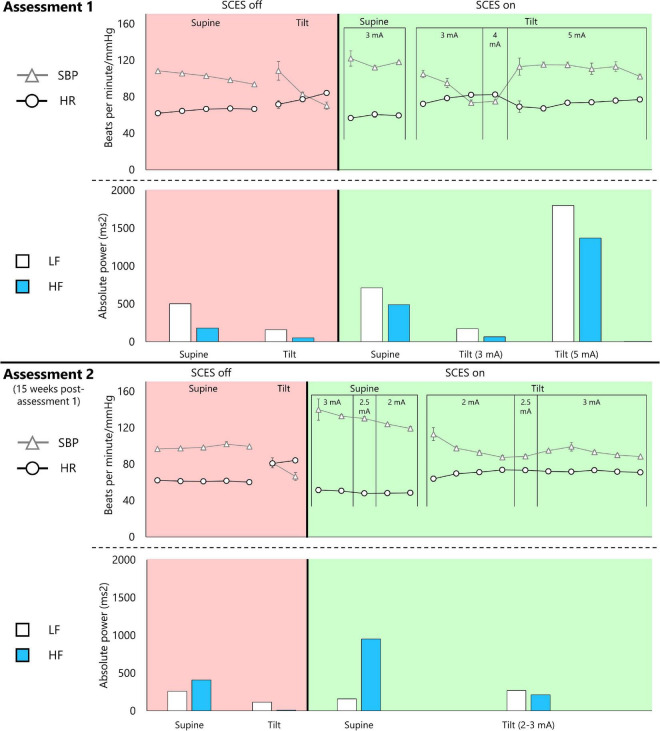
Cardiovascular autonomic response to EAW enhancement SCES during a 45-degree head-up-tilt. Assessment 1 was captured 15 weeks before assessment 2. Both assessments followed the same protocol. The participant was initially in supine lying for 5 min followed by an attempt to perform a head-up tilt using a motorized tilting table for 10 min. The tilting maneuver was discontinued if systolic blood pressure (SBP) dropped by 40 mm Hg from resting baseline. This is followed by repeating the same protocol with SCES on after a 30-min washout period. The SCES was turned on 3–5 min in supine lying before performing the head-up tilt maneuver. A similar timeline was repeated in assessment 2. Triangles and circles represent 1-min averages of SBP and heart rate (HR), respectively. Low-frequency (LF) and high-frequency (HF) components of a heart rate variability analysis are presented for the entire corresponding supine or head-up tilt period.

Detailed results of head-up-tilt testing are presented in Figure 5. At assessment one, without SCES, the participant’s SBP averaged 101.8 mmHg in supine, then dropped rapidly upon tilt, necessitating early test termination. Activating EAW enhancement SCES (3 mA) increased the participant’s supine SBP (average 122 mmHg in minute one), but did not prevent a drop upon tilt–however, increasing the stimulation to 5 mA stabilized SBP above SCES-off supine levels (average 111.5 mmHg), while also yielding the highest LF and HF values.

At assessment two, the participant’s SBP averaged 98.9 mmHg in supine with SCES off. Activating EAW enhancement SCES in supine increased SBP almost twice as much as assessment one (average 140 mmHg in minute one), leading the study team to decrease SCES amplitude to 2 mA. Upon tilt at 2 mA, SBP decreased over 4 min. Increasing SCES amplitude to 3 mA stabilized SBP (average 93.2 mmHg) at values close to that of SCES off supine. 5 mA was not re-tested to avoid further risk of an unwanted increase in SBP. During SCES off, both LF and HF appeared to be attenuated during tilt. However, the HF remarkably dropped during tilt at an amplitude of 3 mA with a modest change in LF.

Thus, at assessment one, EAW enhancement SCES at 3 mA moderately increased SBP in supine (+ 20 mmHg), but did not prevent a drop in SBP upon tilt; whereas at assessment 2, EAW enhancement SCES delivered at 3 mA produced a larger increase in supine SBP (+41 mmHg), yet stabilized SBP during tilt. At both assessments, tonic, non-rhythmic and non-patterned lower extremity muscle activity was induced when SCES was delivered at 3 mA or greater (Figure 4). The amount of muscle activity as measured by EMG is presented for the right and left vastus lateralis muscles (Figure 4).

**FIGURE 6:**
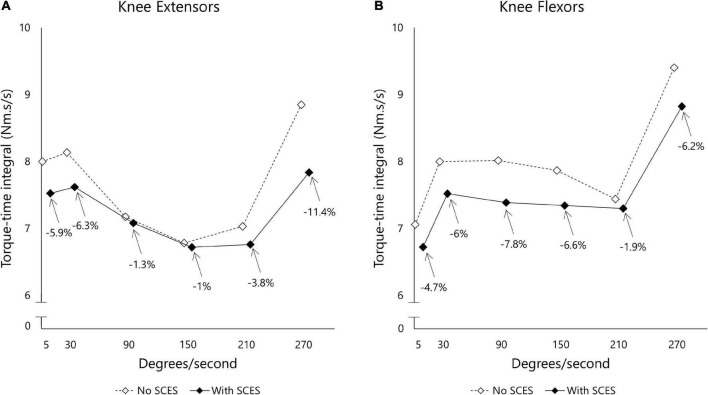
Torque-time integral (Nm.s/s) measured for knee extensor **(A)** and flexor **(B)** muscle groups using isokinetic dynamometer. The examined leg was moved across different angular velocities (5–270 deg.sec^– 1^) and the resistance to passive movement was reported as area under the curve after accounting for the duration (s) of each curve. The task was administered initially with SCES off (open diamond) before a washout period of 30 min in which SCES was turned on (closed diamond).

Figure 6 shows passive torque-time integral measurements obtained at various angular velocities. The induced torque at each angular velocity decreased with SCES on (3 mA), suggesting less resistance to passive movement from the knee flexor (−5.53%) and knee extensor (−4.95%) muscles.

## Discussion

These results show that EAW enhancement SCES produced favorable changes in cardiovascular regulation during an orthostatic challenge, and attenuated resistive torque evoked by passive movement. Thus, the participant’s self-report that this configuration reduced symptoms of low blood pressure and helped with spasticity has merit. When first tested, one amplitude (3 mA) increased SBP in supine but did not prevent a drop in SBP upon tilt. However, after 15 weeks of use during both EAW training and in the participant’s daily life, the same stimulation amplitude caused twice the increase in SBP in supine, yet when tilted stabilized SBP at a value similar to his supine SBP without SCES. Thus, over time, a lower stimulation amplitude was necessary to prevent a drop in SBP upon orthostatic challenge.

The novelty of this case report needs to be highlighted. We have utilized percutaneous SCES implantation and not paddle implantation as has been demonstrated with several other groups (Aslan et al., 2018; Darrow et al., 2019; Ditterline et al., 2020; Squair, 2021; Gorgey et al., 2022). The point is not to emphasize the use of percutaneous leads over paddle implantation but to demonstrate the fact that there is an alternative approach that may yield positive results similar to the paddle. We have noted this point in another patient who had major spinal fusion that would disqualify him from paddle implantation especially with a T11 level of injury (Gorgey et al; unpublished data).

Unlike other reports who specifically stated that performing specific detailed mapping procedures to control for autonomic nervous system (Aslan et al., 2018; Darrow et al., 2019; Ditterline et al., 2020; Squair, 2021). We simply introduced the use of locomotor-like mapping procedure that may help controlling the autonomic nervous system and reduce spasticity; the findings are a step toward making this technology a clinically feasible approach. Unlike researchers, clinicians do not have the time nor the resources for detailed mapping procedures. The findings may indirectly reflect how different nervous system functions are interconnected. A simple rhythmic configuration that facilitated EAW is the same configuration that yielded enhancement of the autonomic functions and reduction in spasticity. Finally assessing spasticity using an isokinetic dynamometer (i.e., Biodex) may provide more quantitative information than simply performing the pendulum test or using the modified Ashworth scale as previously done (Cook, 1976; Richardson et al., 1979; Barolat et al., 1988). This will be a step toward objectively quantifying longitudinal effects of SCES on spasticity after SCI.

The LF and HF bands of a heart rate variability analysis indicate baroreceptor activity and parasympathetic drive, respectively (Shaffer and Ginsberg, 2017). Tilting the participant always reduced the absolute power in the LF and HF bands compared to supine, except when SCES was increased to 5 mA at assessment one. It is noteworthy that LF power was always dominant over HF power at assessment one, even though the absolute values fluctuated depending on the condition. However, in supine at assessment two, whether SCES was on or off, parasympathetic activity seemed dominant as indicated by higher absolute power in the HF band – yet upon tilt LF was dominant, similar to assessment one. Thus, even though tonic muscle activity was induced with stimulation over 3 mA, the changing magnitude and proportion of LF and HF power suggests that SCES-induced muscle activity is not the only factor influencing these cardiovascular findings. Squair (2021) examined in great detail how SCES affected the cardiovascular function. The report demonstrated that SCES can successfully activate the sympathetic circuitries and influence hemodynamics. Other mechanisms may include inducing pressor responses, activation of hypothetical circuities that cause vasoconstriction, and possible activation of interneurons that are connected to splanchnic ganglia. Furthermore, SCES induced immediate baroreflex control of the pressor response in acute injury of three rhesus monkeys after targeting the dorsal left and right roots of T10, T11, and T12 segments despite orthostatic challenges (Squair, 2021). A linear relationship was observed between SCES amplitudes and pressor response which may explain our findings in assessment one when the SCES amplitude was increased from 3 to 5 mA. These neural circuitries may have become more sensitive in assessment two and did not need more than 3 mA to maintain this pressor response.

Exoskeleton-assisted walking enhancement SCES reduced passive torques at the knee by an average of 5.2%. There appeared to be differential effects of SCES for knee extensors vs. flexors at some angular velocities – while the functional implications of this remain unclear, in general, reduced spasticity can allow improved intermuscular coordination and enhance walking, likely through reorganization of spinal circuits. This was shown in previous reports that noted improvements in inter-limb coordination during overground locomotion following SCES (Wagner et al., 2018). Overall, the consistency with which SCES reduced resistive torques induced by passive movement indicates attenuation of unwanted muscle activity at the knee joint, in line with past reports of the benefits of SCES to alleviate spasticity (Pinter et al., 2000).

The effects of different pharmaceuticals and rehabilitation intervention on spasticity after SCI is well studied (Kakebeeke et al., 2005; Sadowsky et al., 2013; McIntyre et al., 2014; Bekhet et al., 2019). A previous systematic review indicated that the use of an intrathecal baclofen pump resulted in reduction in the mean Ashworth scores from 3.1–4.5 at baseline to 1.0 or 2.00 at follow-up that ranged between 2 and 41 months (McIntyre et al., 2014). Another systematic review reported reductions in spasticity by 45–60% following training with electrical stimulation. The study recognized an acute effect of electrical stimulation on spasticity with limited evidence regarding the chronic effect (Bekhet et al., 2019). Sadowsky et al. (2013) measured peak resistance torque of the knee flexors and showed similar results to what we reported in our study following application of functional electrical stimulation for 29 months. Another study that examined the acute effects of passive cycling on spasticity did not detect any difference in peak torques of knee extensors or flexors when using an isokinetic dynamometer (Kakebeeke et al., 2005). Therefore, the acute effect noted in the current report should be replicated in a larger cohort and examined longitudinally to determine the chronic effect which has previously been questioned (Midha and Schmitt, 1998).

Since the mechanism by which EAW enhancement SCES produces these beneficial effects cannot be determined from this case report, a theoretical explanation must be proposed. Walking rehabilitation after SCI without SCES can benefit autonomic cardiovascular regulation (Ditor et al., 2005), spasticity (Mirbagheri, 2015), and even interactions between somato-motor and sympathetic activity (Onushko et al., 2019). These benefits are thought to partially stem from activity in locomotor central pattern generators that walking rehabilitation can elicit (Smith and Knikou, 2016). Conceptually, walking would not be possible without minimizing aberrant neuromuscular activity (i.e., spasticity), or without optimizing systemic circulation (i.e., proper cardiovascular autonomic regulation). In this study, muscle activity induced when SCES was turned on above 3 mA was tonic and non-rhythmic, but this was possibly enough current to stimulate locomotor central pattern generator circuitry enough to enhance the regulation of spasticity and cardiovascular autonomic function. It is also worth noting that similar improvements in autonomic function and spasticity were previously documented when trans-spinal cord stimulation (Rehman et al., 2023) was used with EAW (Gad et al., 2017; Shapkova et al., 2020).

In summary, this case report shows that percutaneously-implanted SCES intended to yield rhythmic locomotor activity and enhance EAW performance resulted in beneficial off-target effects on cardiovascular autonomic function and spasticity in one individual with a chronic motor complete C8 SCI. The current case report may hold promising findings that applications of implanted percutaneous SCES may mitigate several of the chronic disorders after SCI. Furthermore, the use of simple rhythmic configuration may alleviate the burden of detailed mapping procedures, which may preclude feasibility of this approach in clinical settings. Future experimentation is warranted to determine if these results or other multi-use SCES configurations can be developed for other persons with SCI, and to determine the precise mechanisms by which these beneficial off-target effects are generated.

## Patient’s perspective

In 2021, I had the spinal cord epidural stimulator implanted. The programs I have been using greatly help with spasticity and blood pressure. After about an hour with the stimulator on my spasms start to lessen and so on throughout the day until they are gone. My blood pressure is instantly taken to a normotensive level. I have hypotension so the stimulator is able to help drastically. Spasticity and blood pressure are the main effects of using my stimulator and it’s helps with my day-to-day life.

## Data availability statement

The raw data supporting the conclusions of this article will be made available by the authors, without undue reservation.

## Ethics statement

The studies involving human participants were reviewed and approved by the McGuire Research Institute. The patients/participants provided their written informed consent to participate in this study.

## Author contributions

JG contributed to the data collection, data analysis, and manuscript drafting. AA assisted in the data collection. RT participated in the design of the study, the implantation of percutaneous leads, and managing the subject post-operative. AG participated in the design of the study, provided the funding, and contributed to the data collection, and manuscript drafting and editing. All authors contributed to the article and approved the submitted version.

## References

[B1] Angeli C. A. Boakye M. Morton R. A. Vogt J. Benton K. Chen Y. (2018). Recovery of over-ground walking after chronic motor complete spinal cord injury. *N. Engl. J. Med.* 379 1244–1250. 10.1056/NEJMoa1803588 30247091

[B2] Angeli C. Edgerton V. Gerasimenko Y. Harkema S. (2014). Altering spinal cord excitability enables voluntary movements after chronic complete paralysis in humans. *Brain* 137 1394–1409. 10.1093/brain/awu038 24713270 PMC3999714

[B3] Aslan S. C. Ditterline B. E. Park M. C. Angeli C. A. Rejc E. Chen Y. (2018). Epidural spinal cord stimulation of lumbosacral networks modulates arterial blood pressure in individuals with spinal cord injury-induced cardiovascular deficits. *Front. Physiol.* 9:565. 10.3389/fphys.2018.00565 29867586 PMC5968099

[B4] Barolat G. Myklebust J. B. Wenninger W. (1988). Effects of spinal cord stimulation on spasticity and spasms secondary to myelopathy. *Appl. Neurophysiol.* 51 29–44. 10.1159/000099381 3260464

[B5] Beck L. Veith D. Linde M. Gill M. Calvert J. Grahn P. (2021). Impact of long-term epidural electrical stimulation enabled task-specific training on secondary conditions of chronic paraplegia in two humans. *J. Spinal Cord Med.* 44 800–805. 10.1080/10790268.2020 32202485 PMC8477931

[B6] Bekhet A. Bochkezanian V. Saab I. Gorgey A. (2019). The effects of electrical stimulation parameters in managing spasticity after spinal cord injury: A systematic review. *Am. J. Phys. Med. Rehabil.* 98 484–499. 30300228 10.1097/PHM.0000000000001064

[B7] Cook A. W. (1976). Electrical stimulation in multiple sclerosis. *Hosp. Pract.* 11 51–58. 10.1080/21548331.1976.11706516 1088368

[B8] Darrow D. Balser D. Netoff T. I. Krassioukov A. Phillips A. Parr A. (2019). Epidural spinal cord stimulation facilitates immediate restoration of dormant motor and autonomic supraspinal pathways after chronic neurologically complete spinal cord injury. *J. Neurotrauma* 36 2325–2336. 10.1089/neu.2018.6006 30667299 PMC6648195

[B9] Dimitrijevic M. R. Illis L. S. Nakajima K. Sharkey P. C. Sherwood A. M. (1986). Spinal cord stimulation for the control of spasticity in patients with chronic spinal cord injury: II. Neurophysiologic observations. *Cent. Nerv. Syst. Trauma* 3 145–152. 10.1089/cns.1986.3.145 3490313

[B10] Ditor D. Kamath M. MacDonald M. Bugaresti J. McCartney N. Hicks A. (2005). Effects of body weight-supported treadmill training on heart rate variability and blood pressure variability in individuals with spinal cord injury. *J. Appl. Physiol.* 98 1519–1525. 10.1152/japplphysiol.01004.2004 15563629

[B11] Ditterline B. E. Wade S. Ugiliweneza B. Singam N. S. Harkema S. J. Stoddard M. F. (2020). Beneficial cardiac structural and functional adaptations after lumbosacral spinal cord epidural stimulation and task-specific interventions: A pilot study. *Front. Neurosci.* 14:554018. 10.3389/fnins.2020.554018 33192245 PMC7643015

[B12] Gad P. Gerasimenko Y. Zdunowski S. Turner A. Sayenko D. Lu D. (2017). Weight bearing over-ground stepping in an exoskeleton with non-invasive spinal cord neuromodulation after motor complete paraplegia. *Front. Neurosci.* 11:333. 10.3389/fnins.2017.00333 28642680 PMC5462970

[B13] Gill M. L. Grahn P. J. Calvert J. S. Linde M. B. Lavrov I. A. Strommen J. A. (2018). Neuromodulation of lumbosacral spinal networks enables independent stepping after complete paraplegia. *Nat. Med.* 24 1677–1682. 10.1038/s41591-018-0175-7 30250140

[B14] Gorgey A. Gouda J. (2022). Single lead epidural spinal cord stimulation targeted trunk control and standing in complete paraplegia. *J. Clin. Med.* 11:5120. 10.3390/jcm11175120 36079048 PMC9457264

[B15] Gorgey A. Gill S. Holman M. Davis J. Atri R. Bai O. (2020). The feasibility of using exoskeletal-assisted walking with epidural stimulation: A case report study. *Ann. Clin. Transl. Neurol.* 7 259–265. 10.1002/acn3.50983 32023011 PMC7034511

[B16] Gorgey A. Poarch H. Harnish C. Miller J. Dolbow D. Gater D. (2011). Acute effects of locomotor training on neuromuscular and metabolic profile after incomplete spinal cord injury. *NeuroRehabilitation* 29 79–83. 10.3233/NRE-2011-0680 21876299

[B17] Gorgey A. Sutor T. Goldsmith J. Ennasr A. Lavis T. Cifu D. (2022). Epidural stimulation with locomotor training ameliorates unstable blood pressure after tetraplegia. A case report. *Ann. Clin. Transl. Neurol.* 9 232–238. 10.1002/acn3.51508 35068086 PMC8862417

[B18] Harkema S. J. Wang S. Angeli C. A. Chen Y. Boakye M. Ugiliweneza B. (2018). Normalization of blood pressure with spinal cord epidural stimulation after severe spinal cord injury. *Front. Hum. Neurosci.* 12:83. 10.3389/fnhum.2018.00083 29568266 PMC5852107

[B19] Harkema S. Gerasimenko Y. Hodes J. Burdick J. Angeli C. Chen Y. (2011). Effect of epidural stimulation of the lumbosacral spinal cord on voluntary movement, standing, and assisted stepping after motor complete paraplegia: A case study. *Lancet* 377 1938–1947. 10.1016/S0140-6736(11)60547-3 21601270 PMC3154251

[B20] Kakebeeke T. Lechner H. Knapp P. (2005). The effect of passive cycling movements on spasticity after spinal cord injury: Preliminary results. *Spinal Cord* 43 483–488. 10.1038/sj.sc.3101747 15824755

[B21] Kim D. Vakharyia R. Kroll H. Shuster A. (2011). Rates of lead migration and stimulation loss in spinal cord stimulation: A retrospective comparison of laminotomy versus percutaneous implantation. *Pain Physician* 14 513–524. 22086092

[B22] McIntyre A. Mays R. Mehta S. Janzen S. Townson A. Hsieh J. (2014). Examining the effectiveness of intrathecal baclofen on spasticity in individuals with chronic spinal cord injury: A systematic review. *J. Spinal Cord Med.* 37 11–18. 24089997 10.1179/2045772313Y.0000000102 PMC4066544

[B23] Midha M. Schmitt J. (1998). Epidural spinal cord stimulation for the control of spasticity in spinal cord injury patients lacks long-term efficacy and is not cost-effective. *Spinal Cord* 36 190–192. 10.1038/sj.sc.3100532 9554020

[B24] Minassian K. Jilge B. Rattay F. Pinter M. Binder H. Gerstenbrand F. (2004). Stepping-like movements in humans with complete spinal cord injury induced by epidural stimulation of the lumbar cord: Electromyographic study of compound muscle action potentials. *Spinal Cord* 42 401–416. 10.1038/sj.sc.3101615 15124000

[B25] Mirbagheri M. (2015). Comparison between the therapeutic effects of robotic-assisted locomotor training and an anti-spastic medication on spasticity. *Annu. Int. Conf. IEEE Eng. Med. Biol. Soc.* 2015 4675–4678. 10.1109/EMBC.2015.7319437 26737337

[B26] Onushko T. Mahtani G. Brazg G. Hornby T. Schmit B. (2019). Exercise-induced alterations in sympathetic-somatomotor coupling in incomplete spinal cord injury. *J. Neurotrauma* 36 2688–2697. 10.1089/neu.2018.5719 30696387 PMC6727466

[B27] Pinter M. Gerstenbrand F. Dimitrijevic M. (2000). Epidural electrical stimulation of posterior structures of the human lumbosacral cord: 3. Control of spasticity. *Spinal Cord* 38 524–531. 10.1038/sj.sc.3101040 11035472

[B28] Rehman M. Sneed D. Sutor T. Hoenig H. Gorgey A. (2023). Optimization of transspinal stimulation applications for motor recovery after spinal cord injury: Scoping review. *J. Clin. Med.* 12:854. 10.3390/jcm12030854 PMC9917510 36769503

[B29] Rejc E. Angeli C. Harkema S. (2015). Effects of lumbosacral spinal cord epidural stimulation for standing after chronic complete paralysis in humans. *PLoS One* 10:e0133998. 10.1371/journal.pone.0133998 26207623 PMC4514797

[B30] Richardson R. Cerullo L. McLone D. Gutierrez F. Lewis V. (1979). Percutaneous epidural neurostimulation in modulation of paraplegic spasticity. Six case reports. *Acta Neurochir.* 49 235–243. 10.1007/BF01808963 316266

[B31] Sadowsky C. Hammond E. Strohl A. Commean P. Eby S. Damiano D. (2013). Lower extremity functional electrical stimulation cycling promotes physical and functional recovery in chronic spinal cord injury. *J. Spinal Cord Med.* 36 623–631. 10.1179/2045772313Y.0000000101 24094120 PMC3831323

[B32] Sayenko D. Angeli C. Harkema S. Edgerton V. Gerasimenko Y. (2014). Neuromodulation of evoked muscle potentials induced by epidural spinal-cord stimulation in paralyzed individuals. *J. Neurophysiol.* 111 1088–1099. 10.1152/jn.00489.2013 24335213 PMC3949232

[B33] Shaffer F. Ginsberg J. (2017). An overview of heart rate variability metrics and norms. *Front. Public Health* 5:258. 10.3389/fpubh.2017.00258 29034226 PMC5624990

[B34] Shapkova E. Pismennaya E. Emelyannikov D. Ivanenko Y. (2020). Exoskeleton walk training in paralyzed individuals benefits from transcutaneous lumbar cord tonic electrical stimulation. *Front. Neurosci.* 14:416. 10.3389/fnins.2020.00416 32528238 PMC7263322

[B35] Smith A. Knikou M. A. (2016). Review on locomotor training after spinal cord injury: Reorganization of spinal neuronal circuits and recovery of motor function. *Neural Plast.* 2016:1216258. 10.1155/2016/1216258 27293901 PMC4879237

[B36] Squair J. (2021). Neuroprosthetic baroreflex controls haemodynamics after spinal cord injury. *Nature* 590 308–314. 10.1038/s41586-020-03180-w 33505019

[B37] Wagner F. B. Mignardot J. Goff-Mignardot C. G. Demesmaeker R. Komi S. Capogrosso M. (2018). Targeted neurotechnology restores walking in humans with spinal cord injury. *Nature* 563 65–71. 10.1038/s41586-018-0649-2 30382197

[B38] West C. R. Phillips A. A. Squair J. W. Williams A. M. Walter M. Lam T. (2018). Association of epidural stimulation with cardiovascular function in an individual with spinal cord injury. *JAMA Neurol.* 75 630–632. 10.1001/jamaneurol.2017.5055 29459943 PMC5885254

